# The Role of Neutrophils in ANCA-Associated Vasculitis: The Pathogenic Role and Diagnostic Utility of Autoantibodies

**DOI:** 10.3390/ijms242417217

**Published:** 2023-12-07

**Authors:** Agata Walulik, Kinga Łysak, Michał Błaszkiewicz, Ignacy Górecki, Krzysztof Gomułka

**Affiliations:** 1 Student Scientific Group of Adult Allergology and Internal Medicine, Wroclaw Medical University, 50-369 Wrocław, Poland; agata.walulik@gmail.com (A.W.); ignacygorecki@gmail.com (I.G.); 2 Faculty of Medicine, Medical University of Gdansk, 80-210 Gdańsk, Poland; kinga.lysak@op.pl; 3 Department of Internal Medicine, Pneumology and Allergology, Wroclaw Medical University, 50-369 Wrocław, Poland

**Keywords:** neutrophil, antineutrophil-cytoplasmic antibodies (ANCAs), vasculitis, proteinase 3-/ myeloperoxidase-ANCA, ANCA-associated vasculitis (AAV)

## Abstract

Recent years have brought progress in understanding the role of the neutrophil, dispelling the dogma of homogeneous cells mainly involved in the prime defence against pathogens, shedding light on their pathogenic role in inflammatory diseases and on the importance of antineutrophil-cytoplasmic antibodies’ pathogenic role in ANCA-associated vasculitides vasculitis (AAV). Myeloperoxidase (MPO) and proteinase 3 (PR3) expressed in neutrophil granulocytes are the most common targets for ANCAs and contribute to the formation of MPO-ANCAs and PR3-ANCAs which, released to the bloodstream, become an excellent diagnostic tool for AAV. In this study, we focus on increasing the clinical and experimental evidence that supports the pathogenic role of ANCAs in AAV. Additionally, we discuss the diagnostic utility of ANCAs for disease activity and prognosis in AAV. Understanding the central role of ANCAs in AAV is crucial for advancing our knowledge of these complex disorders and developing targeted therapeutic strategies in the era of personalized medicine.

## 1. Search Strategy

For critical parts of our review, the PubMed and Google Scholar databases were searched for the key words. The time limit for included articles was set from 2010 to 2023.

## 2. Introduction

Antineutrophil-cytoplasmic-antibody-(ANCA)-associated vasculitis (AAV) is classified as a “primary vasculitis”, based on the most widely used classification of AAV at the 2012 international Chapel Hill consensus conference (CHCC). This term is often used to describe medium- and small-vessel disease (i.e., capillaries, venules, arterioles, and small arteries). It differentiates vasculitis occurring de novo from secondary vasculitis connected to infections, connective-tissue diseases or some types of malignancy [1]. AAV is a group of diseases, which clinically involves many organ systems, including the upper- and lower-respiratory tracts, kidneys, skin, and nerves [2].

AAV diseases comprise granulomatosis with polyangiitis (GPA), microscopic polyangiitis (MPA), and eosinophilic granulomatosis with polyangiitis (EGPA) [2]. The two most important ANCA antigens are proteinase 3 (PR3) and myeloperoxidase (MPO) [3]. The presence of PR3-ANCA is highly suggestive (80%) of granulomatosis with polyangiitis (GPA), while MPO-ANCA is associated (70%) with microscopic polyangiitis (MPA). Approximately 60% of EGPA is negative for both PR3- and MPO-ANCA [4,5]. The disease relations of PR3-ANCA and MPO-ANCA are provided below in Table 1.

The incidence of AAV has risen over time. This could be attributed to advancements in ANCA (antineutrophil-cytoplasmic antibody) testing, better disease-classification methods, and improved clinical recognition. AAV affects males and females equally. This implies that there is no significant gender predilection for the disease [6]. Patients with GPA and MPA, which are the most common clinical phenotypes of AAV, are typically older adults. By contrast, patients with eosinophilic GPA (EGPA) tend to be younger. This may be linked to its close association with asthma [7,8]. The incidence of AAVs varies by region and specific subtypes. The majority of studies on GPA and MPA have been conducted in countries where most patients are of European descent, leading to a higher prevalence of GPA compared to MPA. However, in Japan and China, MPA is more common than GPA. When comparing observational cohorts of GPA and MPA patients between Japan and the United Kingdom, several differences have been observed, including a greater prevalence of MPO-ANCA positivity, variations in disease manifestations, differences in the distribution of sexes, and variations in the age of onset [9]. EGPA has the lowest global incidence (1.7 per million person-years), GPA has the highest global incidence rate of 96.8 per million, and MPA has a global incidence rate of 39.2 per million [10].

Over the past few years, there has been a gradual increase in the number of individuals being diagnosed with AAV. Various reasons have been suggested to account for this increase, including shifts in climate patterns, enhanced diagnostic criteria, more widespread ANCA testing, and heightened awareness of the condition [11,12].

## 3. Vasculitis

### 3.1. Historical Perspective and Development of Subject’s Common Understanding

Although vascular diseases in a broad sense were known of in ancient times, the term “vasculitis” in the context of a distinct disease entity has only been used for about 150 years. Since then, there has undoubtedly been significant progress in the diagnosis, treatment, and classification of these disorders [13].

In 1931, Heinz Klinger was the first to describe the disease now called granulomatosis with polyangiitis. He presented the case history of a 70-year-old patient with symptoms of kidney inflammation, joint inflammation, and chronic sinusitis [14]. A few years later, in 1939, Friedrich Wegener published three similar cases of patients. Histopathological examination conducted by him revealed dominating granulomatous-necrotic-inflammatory changes in the respiratory tract, necrotising vasculitis, and necrotising glomerulonephritis. He described this entity as a “peculiar rhinogenous granulomatosis with a unique participation of the arterial system and the kidneys” [15].

Gabriel Godman and Jacob Churg recognised Wegener’s contribution to understanding the condition in 1954 by using the term “Wegener’s Granulomatosis” (WG) [16]. They established the classical diagnostic criteria of WG. The Wegener triad included granulomas, vasculitis, and glomerulonephritis [17]. Increased awareness of Wegener’s association with and potential involvement in the Nazi regime led to a shift in the clinical and academic community’s perspective. This shift prompted a change in terminology, specifically the removal of the eponymous name “Wegener’s” in favour of the term “granulomatosis with polyangiitis”. This terminology change was officially adopted during the 2012 revision of the Chapel Hill consensus conference on the nomenclature of vasculitis.

In 1985, a Dutch–Danish group described auto-antibodies against granulocyte- intracytoplasmic antigens (termed as ANCAs—antineutrophil-cytoplasmic antibodies) as a specific marker for WG. The diagnostic importance of ANCAs was soon universally accepted and enabled also a new understanding of a whole disease group termed “systemic vasculitis”, especially diseases like Wegener’s granulomatosis, microscopic polyangiitis, and Churg–Strauss syndrome [18].

### 3.2. Current Classification Criteria for Systemic Vasculitis

The classification of systemic vasculitis is a complex process that is based on a combination of clinical features and pathological findings. The first systematic approach began with the work of Pearl Zeek in 1952 and the American-College-of-Rheumatology-(ACR) classification in 1990 [19].

The two main systems of classification that are commonly used to categorise systemic vasculitis based on the size of the primarily affected blood vessels are the rheumatology (ACR) classification criteria, developed in 1990, and the Chapel Hill consensus definitions (CHCC) from 1994, revised in 2012. These systems help healthcare professionals diagnose and manage vasculitic conditions more effectively [2].

There have been significant advancements in the methodology for developing classification criteria for vasculitis. The diagnostic and classification criteria in vasculitis study (DCVAS) was established to create contemporary and practical classification criteria for major systemic vasculitis. A new classification has recently been proposed by ACR–EULAR. The specificity and sensitivity of the ACR–EULAR criteria are better than those of the ACR 1990 criteria, as they take into account ANCAs and modern imaging techniques [20]. In the current classification, additionally, secondary vasculitis is identified [21]. Among the causes of secondary vasculitis, infections, medications, systemic-connective-tissue diseases, and malignant tumours are mentioned [22].

The first level of classification of primary systemic vasculitis relates to the predominant type of vessels involved—inflammation of large, medium, or small vessels [2]. Large-vessel vasculitis (LVV) includes Takayasu arteritis (TA) and giant-cell arteritis (GCA). Inflammation of medium-sized vessels (MVV) includes classical polyarteritis nodosa (PAN) and Kawasaki disease (KD) [23,24]. Two forms of small-vessel vasculitis (SVV) are distinguished:

1. Vasculitis associated with antineutrophil-cytoplasmic antibodies-(ANCA-associated vasculitis; AAV) [25].

2. Vasculitis associated with immunologic complexes—anti-glomerular-basement-membrane-(anti-GBM) disease, cryoglobulinemic vasculitis (CV), IgA vasculitis, and hypocomplementemic-urticarial vasculitis (HUV) [26]. The current AAV classification is presented in Table 2.

## 4. The Neutrophil

Neutrophils are the most abundant cell population among leukocytes, comprising 50–70% of the white-cell pool [29,30]. They are generated in bone marrow through the process of granulopoiesis. The number of cells can vary, depending on factors such as infection, inflammation or stress. In healthy individuals, there are about 1800–8000 neutrophils per microlitre of blood in circulation, with about 5 × $10^{10}$–10 × $10^{10}$ neutrophils produced in the steady state [30,31]. The body continuously produces and replaces neutrophils to maintain this range, but in cases of infection, production can boost by up to tenfold [29].

Even though their essential role is the first line of defence against pathogens including bacteria, fungi, and protozoa in the mechanism of non-specific immunity, they also play a significant role in acute inflammation as one of the first responders [32,33]. For many years, neutrophils were considered a short-living, homogeneous group of cells specialising in fighting pathogen exposure, but studies in recent years have shown that neutrophils, through their phenotypic distinctiveness, are involved in many pathological processes, including cancer, infections, and autoimmune diseases [33].

### Neutrophil Development and Brief Characteristics

**Figure 1:**
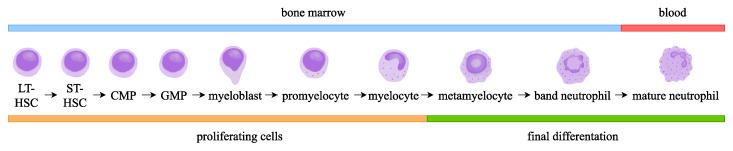
Neutrophil development in bone marrow, detailing the localisation and characteristics of each cell among granulopoiesis.

Granulopoiesis is located in the venous sinuses in the bone marrow and is initiated by common myeloid progenitors (CMPs), which are derived from haematopoietic stem cells (HSCs) [34,35]. At this stage, myeloid-progenitor cells have the ability to differentiate into the following pathways: thrombopoiesis, erythropoiesis, monocytopoiesis, formation of mast cells, and granulopoisesis [36,37]. The first cell of the myeloid lineage is a granulocyte–monocyte progenitor (GMP)—an oligopotent progenitor that during the following stage differentiates into unipotent-neutrophil-committed-myeloblast cells [29,35]. From this stage, the cells differentiate into the final form of mature neutrophils through subsequent differentiation stages: promyelocytes, myelocytes, metamyelocytes, and, finally, band neutrophils [29,30,34,35,38]. The development of the neutrophil is depicted in Figure 1.

Granulopoiesis is driven primarily by the granulocyte-colony-stimulating factor (G-CSF); the cytokines interleukin (IL)-6, IL-4, and the granulocyte-macrophage colony-stimulating factor play a secondary role to neutrophil synthesis [29].

Within the bone marrow, the neutrophil population is divided into three compartments: (1) the stem-cell pool; (2) the mitotic pool; and (3) the post-mitotic pool [31,35]. The stem-cell pool refers to undifferentiated progenitor cells, the mitotic pool consists of intensive and rapidly proliferating and differentiating neutrophil-committed progenitors, while the post-mitotic pool is composed of fully differentiated neutrophils forming an extensive reserve to be released [31,38].

The granules of neutrophils are developed during granulopoiesis from the early promyelocyte stage and can be traditionally divided into two types: (1) peroxidase-positive granules, also called azurophil granules; (2) peroxidase-negative granules [39]. Granule synthesis occurs sequentially, and the expression of the proteins contained in the granules is limited to particular stages of granulopoiesis. Disruption of the timing of protein expression leads to the packaging of proteins into the wrong type of granules and, thus, to heterogeneity within the granules [40].

The major protein of peroxidase-positive granules is myeloperoxidase (MPO), which mainly catalyses the formation of reactive oxygen intermediates in the condition of microbial invasion [39,41]. Other azurophil-granules proteins are proteases, cathepsin G, neutrophil elastase, and the enzymatic inactive proteases CAP37, NSP4, and proteinase 3 (PR3) [39,42]. The last one is an autoantigen with multifaceted properties. It possesses catalytic activity, serves as a haematopoietic regulator, and exhibits apoptosis-inducing capabilities.

## 5. Pathogenesis and Influence of Neutrophilic Elements on the Development of Vasculitis

While the various phenotypic manifestations of AAV exhibit common features, recent research has indicated notable distinctions in the pathogenic mechanisms, involving both the adaptive and innate immune systems. These conditions are typically multifactorial, involving a combination of environmental, genetic, and immunological factors [43].

In the pathomechanism of AAV, both T and B lymphocytes play relevant roles. Infectious antigens are presented by dendritic cells to naive T cells, which differentiate into T helper 17 and produce IL-17 A. Macrophages stimulated by IL-17 A produce pro-inflammatory cytokines, such as TNF and IL-1β. They activate neutrophils and express target antigens on their cell surface. Meanwhile, the alternative-complement pathway also activates neutrophils via binding C5a to its surface receptors [44].

At the same time, neutrophils stimulated by antigens form neutrophil-extracellular traps (NETs) [44]. MPO and PR3 antigens are released into the extracellular environment when neutrophils undergo apoptosis following the release of NETs. These antigens are phagocytosed by antigen-presenting cells, initiating the activation of MPO- and PR3-specific CD4+ T lymphocytes, which are activated within the peripheral blood [45]. Th2 cells support B-cell activity, aiding in the production of ANCA antibodies. They are their primary source in AAV. Plasma cells then start producing ANCA antibodies, which are released into the bloodstream. They bind to neutrophils, especially in the small blood vessels. This triggers a cascade of inflammatory reactions, leading to the activation of neutrophils, which release damaging substances such as ROS and lytic enzymes and contribute to the inflammation seen in AAV [44,46,47]. Moreover, a significant contribution to tissue damage is connected with the further formation and release of neutrophil-extracellular traps (NETs) associated with neutrophil apoptosis (NETosis). A recent study revealed that ANCA-induced NET formation is controlled by receptor-interacting protein kinase (RIPK) 1/3- and mixed-lineage kinase domain-like (MLKL), key mediators of the necroptosis pathway [48]. The pathway controlling NET formation in response to ANCAs remains unclear. NETs in AAV were implicated in promoting autoimmunity by presenting ANCA antigens and contributing to endothelial injury. The study revealed a decrease in NET-forming cells and NET area with RIPK1 and MLKL inhibition [48].

Kimura and colleagues developed a mouse model of MPA, using a unique extract from Candida albicans and by genetically eliminating phosphoinositide 3-kinase γ (PI3K-γ) [49]. In this model, they observed the accumulation of NETs in vivo, increased levels of ANCAs, small-vessel vasculitis, and crescentic glomerulonephritis. Blocking PI3K-γ reduced these abnormalities, suggesting its potential as a therapeutic agent. PI3K-γ is involved in neutrophil chemotaxis and ROS generation [50]. Blocking the C5a receptor alone prevented neutrophil infiltration into glomeruli and crescent formation in ANCA-induced glomerulonephritis [51]. Targeting PI3K-γ may be a viable option for treating patients with MPA.

According to current studies, it has been suggested that intravenous immunoglobulin (IVIG) therapy has a positive effect in patients with MPO-ANCA vasculitis. Results based on rat models show that human intravenous sulfo-immunoglobulins (IVIG-S) inhibit NET formation, suppressing MPO-ANCA production and the development of vasculitic lesions [52]. Furthermore, IVIG-S induce neutrophils lactoferrin secretion, which is known to be an endogenous regulator of NET formation [52,53,54].

NETs are extracellular fibrillar structures containing DNA, serving as a crucial defence mechanism of neutrophils against extracellular pathogens [55]. NETs contain pro-inflammatory proteins and contribute to vascular inflammation by activating the complement system and damaging endothelial cells [47,56]. In the process of NET formation, the substances found in neutrophilic granules, such as MPO and PR3, become mixed with chromatin fibres and adhere to DNA [57]. PR3- and MPO-ANCAs activate the complement system and indirectly contribute to vessel inflammation by acting as a link between the innate and adaptive immune systems [47]. This interaction could potentially alter the immunogenicity or antigenicity of these autoantigens. Moreover, some research shows that excessive exposure to NETs can cause angiopathy [48,58].

**Figure 2:**
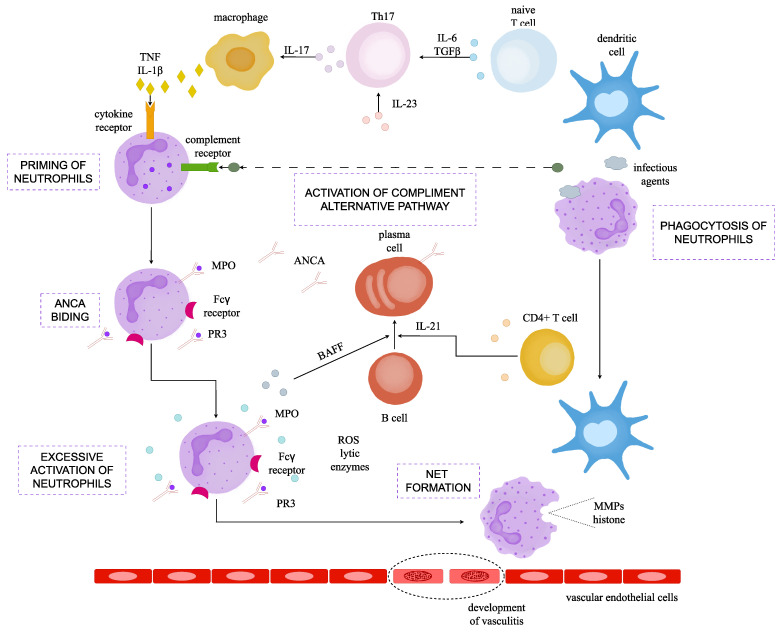
Pathomechanism of NET formation and AAV development. Dendritic cells present antigens to naive T cells. Consequently, these T cells differentiate into T-helper-17-(Th17) cells stimulated by IL-6, TGFβ, and IL-23, and then produce interleukin-17 (IL-17). Subsequently, macrophages, under the influence of IL-17, generate cytokines like tumour necrosis factor (TNF) and interleukin-1β (IL-1β), thereby priming neutrophils for action. Simultaneously, the activation of the alternative complement pathway leads to neutrophil priming. In response to infectious stimuli, neutrophils form neutrophil extracellular traps (NETs). In individuals with compromised NET-degradation activity, persistent NETs lead to a breakdown in tolerance to specific self-antigens, notably myeloperoxidase (MPO) and proteinase 3 (PR3). Dendritic cells subsequently present these self-antigens to CD4+ T cells, triggering the production of antineutrophil-cytoplasmic antibodies (ANCAs) by B cells. Primed neutrophils express MPO and PR3 on their cell surface, binding to PR3-ANCAs and MPO-ANCAs. Concurrently, the Fc region of these ANCAs binds to the Fcγ receptor on the neutrophils, inducing excessive activation. This results in abnormal cytokine production, accompanied by the release of reactive oxygen species (ROS), lytic enzymes, and further NET formation, causing damage to vascular endothelial cells. Noteworthy angiopathic molecules found in NETs include histones dissociated from DNA and matrix metalloproteinases (MMPs). B-cell-activating factor (BAFF or TNF), produced by activated neutrophils and CD4+ T cells via IL-21 stimulate B cells, fostering continuous production of ANCAs [44].

On the other hand, in individuals with a genetic predisposition, combined with the influence of environmental factors, pro-inflammatory cytokines stimulate neutrophils to display MPO and PR3 antigens on their cell surfaces, making them visible to autoreactive cells from the adaptive immune system [11]. These antigens can then become targets for ANCA antibodies. These antibodies, in turn, further activate circulating neutrophils, promoting their transmigration through the endothelial layer and accumulation within the vascular wall. Here, they release reactive oxygen species (ROS) and oxygen enzymes, molecules which cause vessel necrosis [59]. The pathomechanism of NET formation and AAV development is depicted in Figure 2.

Understanding the complex AAV pathomechanism is vital for better management of therapeutic processes and for assessing its impact on disease progression. Therapeutic advancements in ANCA-associated vasculitis over the past two decades have been significant. Based on the pathophysiological differences and similarities, PR3-AAV and MPO-AAV are treated differently. Induction–remission therapy should consist of a combination of corticosteroids and either cyclophosphamide or rituximab [60]. Prior to the adoption of high-dose glucocorticoids (GC) and cyclophosphamide (CYC), the mortality rate for individuals diagnosed with severe AAV was as high as 80% within one year of diagnosis [61]. However, recent improvements have led to a notable reduction in mortality rates. The estimated five-year survival rates are now between 74–91% for granulomatosis with polyangiitis (GPA) and 45–76% for microscopic polyangiitis (MPA). Currently, the most renowned treatment for AAVs is rituximab. Rituximab is a monoclonal anti-CD20 antibody that depletes B cells. Rituximab has not only established itself as the new standard for initiating treatment but has also demonstrated superiority over other agents in the maintenance phase [62]. The understanding of the immunopathogenesis of AAVs has increased in recent decades. Specific biomarkers that distinguish specific types of ANCA-associated vasculitis have been identified, and further research in this field is on the way. Discoveries related to immunopathogenesis translated into clinical practice as targeted therapies are on the rise. As the alternative complement pathway is crucially involved in the pathogenesis of ANCA-associated vasculitis, CCX168 (Avacopan) treatment is likely to be approved as steroid-sparing in the induction of remission of ANCA-associated vasculitis [63]. Another example of routine use in the management of ANCA-associated vasculitis is tocilizumab, a monoclonal antibody targeting the IL-6 receptor. A recent review of the literature identified 17 cases who had received tocilizumab in the management of ANCA-associated vasculitis. A majority (88.2%) achieved remission after IL-6R blockading with tocilizumab [64]. Such examples in the treatment of ANCA-associated vasculitis with positive results can lead to better management of these potentially devastating diseases.

## 6. Antineutrophil-Cytoplasmic Antibodies

Antineutrophil-cytoplasmic antibodies (ANCAs) are a family of auto-antibodies, primarily of the IgG type, that target antigens found in cytoplasmic granules of polymorphonuclear neutrophil granulocytes (PMNs) [65]. They are particularly associated with a group of disorders known as ANCA-associated vasculitis (AAV), which involves systemic vasculitis [20]. The two major types of ANCAs have different cellular localisation patterns. One type is associated with staining around the nucleus (p-ANCA), whereas the other type is associated with diffuse staining of the cytoplasm (c-ANCA). In addition to these two types, there are other patterns recognised and often referred to as atypical ANCA (a-ANCA) [66].

## 7. Diagnostics

**Figure 3:**
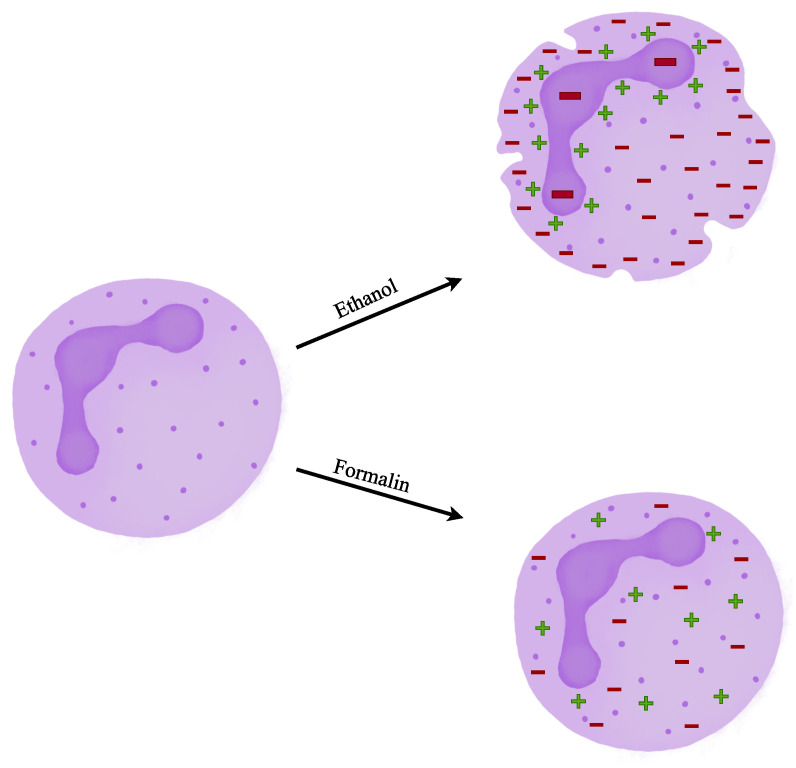
Association between IIF staining pattern and used fixative [78].

Around 90% of patients with multisystemic active GPA have ANCAs positively, and the same applies to cases of MPA [67,68]. Types PR3- and MPO-ANCA are associated with EGPA with variable frequencies. A distinguishing feature of EGPA is the presence of eosinophilia in both peripheral blood and affected tissues. The absence of eosinophilia should raise concerns and lead to a re-evaluation of the diagnosis [69]. In addition to PR3 and MPO, ANCAs can target several other molecules derived from neutrophils. These include cathepsin G, lactoferrin, elastase, defensin, α-enolase, moesin, azurocidin, bactericidal-permeability-increasing protein (BPI) and lysosome-associated membrane glycoprotein 2 (LAMP2) [70,71,72]. The pathogenicity of these “minor” ANCAs is generally considered to be low, and p-ANCA patterns other than MPO-ANCA are typically not associated with vasculitis [73,74]. ANCA testing focuses on two main techniques: indirect immunofluorescence (IIF) on fixed neutrophils and specific PR3- and MPO-ANCA solid-phase immunoassays [75,76]. We can observe three main fluorescence patterns: perinuclear (p-ANCA), cytoplasmic (c-ANCA), and atypical (a-ANCA) [4,66,76,77]. Typically, anti-PR3 antibodies result in a cytoplasmic (c-ANCA) pattern, while anti-MPO antibodies tend to display a perinuclear (p-ANCA) pattern, although there are occasional exceptions [3,76]. Both target antigens are located in the azurophile (primary) granules of neutrophils. They can be exposed on the cell surface and can be excreted through the inflammatory process [4]. In the IIF method, the neutrophils are fixed to the field with ethanol or formalin [75,78]. However, differences in substrates, microscope settings or fixatives may lead to different staining patterns. Ethanol dissolves the lipid barrier of the cytoplasmic granules, which enables positively charged antigens to arrange around the negatively charged nucleus. Formalin fixation leaves the antigens inside the cytoplasmic granules and promotes the c-ANCA pattern [78]. The fixatives impact is presented in Figure 3.

According to the 1999 international consensus, the method of choice for ANCA standard screening was IIF with ethanol fixation [78,79,80]. Each type of staining pattern suggests the presence of the particular diseases [78,81,82], as presented in Table 3.

Current research shows that IIF ANCA testing is characterised by sensitivity of approximately 84%, specificity of 91%, and accuracy of 0.874 [83,84]. Fully automatic IIF-staining-pattern recognition has 95% agreement with visual IIF. This technique can help to standardise the procedure and eliminate subjective image interpretation. According to performed studies, analysed automatic IIF systems were characterised by a total sensitivity rate of approximately 96.7% and a specificity rate of 89.9% [84,85].

The EUVAS multicentre study described the specificity of antigen-specific immunoassays as 98–99% PR3-ANCA, which was higher than the MPO-ANCA (96–99%). For IIF, the specificity of C-ANCA ranged from 97% to 98%, surpassing the specificity of P-ANCA, which ranged from 81% to 96%. The sensitivity of IIF was, respectively, 64% to 78% (C-ANCA) in GPA patients and 85% to 89% (P-ANCA) in MPA patients. For reference, the sensitivity of specific immunoassays was 75% to 80% (PR3-ANCA) in the GPA group and 68% to 86% for MPO. The AUC of the ROC for discriminating between AAV and controls was 0.923 for one IIF method and 0.843 for the other. In the case of antigen-specific immunoassays, the AUC ranged between 0.936 and 0.959. The accuracy for antigen-specific immunoassays ranged between 0.944–0.954. ROC curve analysis revealed a significant advantage of antigen-specific immunoassays, which definitely surpassed IIF, especially in its specificity. Moreover, the authors suggested that primary ANCA screening should focus on PR3 and MPO-specific assays [86].

In another study, utilising chemiluminescence (Bioflash, Werfen; Bedford, MA, USA), the investigation identified an anti-MPO-antibody cut-off point of 41.5 IU/mL for the diagnosis of vasculitis, with an area under the curve (AUC) of 0.8084. Moreover, an analysis focused specifically on diagnosing microscopic polyangiitis established a cut-off point of 36.5 IU/mL, with an AUC of 0.6435. The diagnostic threshold for anti-proteinase 3 (anti-PR3) in vasculitis was established at 20.5 IU/mL, yielding an AUC of 0.7318. Unfortunately, this study did not present an analysis restricted specifically to granulomatosis with polyangiitis. Furthermore, no differences were identified between patients who experienced a more severe disease progression and those who did not [87].

**Figure 4:**
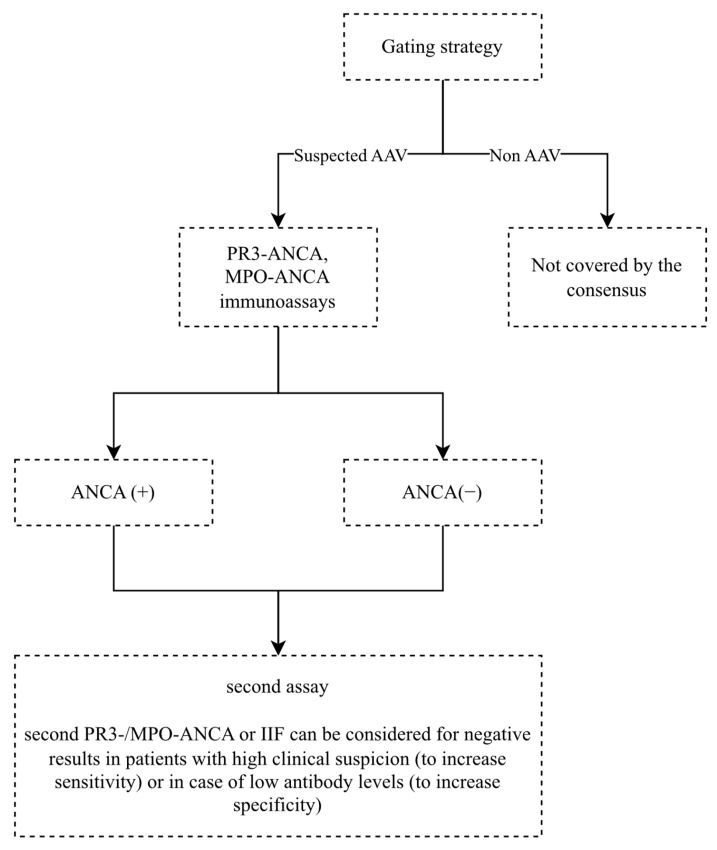
Current ANCA diagnostic recommendations in AAV [88].

According to the international consensus, to confirm the diagnosis ideally both IIF and antigen-specific immunoassays should be performed on each sample [80]. However, the consensus was revised in 2017, and now specific immunoassays are recommended for primary ANCA screening in AAV [88]. Current ANCA diagnostic recommendations have been presented in Figure 4.

The ANCA negative test nonetheless cannot exclude the AAV diagnosis. One of the potential challenges in ANCA testing may be antigens’ epitopes reactivity [86].

The PR3 gene is situated on chromosome 19p13.3, forming a genetic cluster alongside human leukocyte elastase (HLE) and azurocidin, encompassing a genomic span of 6570 base pairs, consisting of five exons and four introns [89]. The gene is transcribed in the promyelocytic stage. In the translation process, PR3 emerges as a prepro-enzyme and subsequently undergoes a series of four enzymatic steps, culminating in the production of the mature form comprising 222 amino acids [90]. Introns I and IV contain repetitive motifs, which have the potential to induce chromosomal instability and may influence genetic rearrangements and deletions [91]. ANCAs have an affinity not to an aminoacid sequence but to multiple diverse conformational epitopes of the target antigens [4,92]. However, approximately 10% of patients with small-vessel vasculitis clinically test negative for ANCA [93]. Diverse subgroups with specific conformation sensitiveness to MPO-ANCA and PR3-ANCA have different associations with disease severity, which may potentially clarify contentious differences between clinical manifestations and laboratory findings [4,92]. Although ANCA negativity does not exclude AAV diagnosis, diffuse AAV forms typically coincide with positive ANCA serum levels [94].

Ideally, the diagnosis AAV is confirmed with a biopsy. The selection of the biopsy site depends on the patient’s clinical presentation. Commonly preferred biopsy sites include the skin, kidneys, temporal artery, muscle, nasal mucosa, lungs, sural nerve, and testes. However, histological examinations can sometimes appear normal (yielding positive results in only approximately 45% of cases involving sural nerve biopsies in patients with confirmed vasculitis) or may reveal only non-specific findings [95].

Among patients with clinical suspicion of systemic vasculitis, laboratory blood tests can also give useful diagnostic information. The most important laboratory panel includes complete blood count, renal function, liver function, and inflammatory markers [95].

Imaging evaluation also has greatly developed in the diagnosis of AVV in recent times. It represents a fundamental step in the diagnostic approach and monitoring processes of vasculitis. It provides the potential for early detection of vessel inflammatory changes and can indicate the disease extension. Imaging procedures identify complications, such as stenosis, occlusion, and aneurysm, and prove valuable in the evaluation of therapeutic response [96].

Diagnosing vasculitis requires high suspicion, due to its rarity and multisystemic nature. A comprehensive evaluation, including a detailed medical history and physical examination, plays a crucial role in managing vasculitis. It is essential to conduct a thorough workup to establish the diagnosis, rule out alternative causes, and assess the disease’s severity and organ involvement. Diagnosis typically involves a combination of clinical assessment, serological testing, radiologic presentations, and histopathological examinations [97].

## 8. Conclusions and Future Prospects

AAV can present numerous diagnostic challenges. Diagnosis of AAV is often delayed or missed because patients may exhibit diverse clinical symptoms, spanning from isolated cutaneous vasculitis to multi-systemic involvement. Also, these diseases lack pathognomonic features [95]. Currently, there are no validated diagnostic criteria for AAV, and the vasculitis classification scores are designed for classification rather than diagnosis of the disease. Diagnosing vasculitis typically involves a combination of clinical assessment and serological testing, with tissue biopsy often serving to confirm the diagnosis [97].

ANCAs have promising potential in the diagnostics process, assessing therapy efficacy and even predicting the disease onset and staging [98,99]. The prevailing evidence suggests focusing on ANCA-specific immunoassays development, due to their high sensitivity and specificity compared to results based on IIF. ANCAs seem to play a crucial role in AAV and can be helpful in the diagnosis of many connective-tissue diseases, such as idiopathic interstitial pneumonia, autoimmune liver diseases, inflammatory bowel diseases, anti-glomerular-basement-membrane-(GBM) disease, and even in some infections or malignancy [79].

We observed a lack of current studies about target antigens, especially MPO and its role in AAV onset and development. A better understanding of AAV pathogenesis can improve the diagnostic process and therapeutic management. New treatment strategies can target inhibition of necroptosis-induced kinases, such as RIPK1/3 and MLKL [48].

Further research in the field of AAV and ANCAs is essential to enhancing our understanding of these conditions and to developing more accurate and timely diagnostic criteria. Improved diagnostic tools can have a significant impact on patient outcomes, enabling early intervention and tailored treatment strategies. Additionally, a deeper exploration of the role of ANCAs in various autoimmune and inflammatory diseases may open up new avenues for the development of targeted therapies and more comprehensive disease management strategies. 

## Figures and Tables

**Table 1:** Disease relations of PR3- and MPO-ANCA.

	PR3 (%)	MPO (%)	Negative (%)
GPA	80	15	5
MPA	20	70	10
EGPA	5	35	60

**Table 2:** ANCA-associated vasculitis [6,27,28].

Feature	GPA	MPA	EGPA
Epidemiology	130 cases/million	20 cases/million	1–3 cases/million
ANCA(+)	80–90%	60–80%	30–40%
ANCA antigen specificity	PR3 > MPO	MPO > PR3	MPO > PR3
Histology	Granulomatous inflammation, tissue necrosis, small–medium-vessel vasculitis, renal-biopsy focal-segmental-necrotising glomerulonephritis with crescent formation, skin-biopsy leukocytoclastic vasculitis	Leukocytoclastic vasculitis, no granulomatous inflammation	Tissue eosinophilia, necrotising vasculitis, eosinophil-rich granulomatous inflammation
Upper-respiratory-tract involvement	Nasal septal perforation, saddle-nose deformity, conductive or sensorineural hearing loss, subglottic stenosis (90%)	Absent or mild	Nasal polyps, allergic rhinitis, conductive hearing loss (70%)
Lower-respiratory-tract involvement	Nodules, infiltrates, or cavitary lesions, alveolar haemorrhage, cough, shortness of breath, chest pain (50–90%)	Shortness of breath, exertion dyspnoea, cough, expectoration of sputum with blood, alveolar haemorrhage (10–30%)	Asthma (>95%), fleeting infiltrates, alveolar haemorrhage
Cardiac involvement	Granulomas, effusion of pericardium, pericarditis, myocarditis, arrhythmias, increased risk of coronary artery disease and acute coronary syndrome, congestive heart failure (6–44%)	Pericarditis, cardiac failure (3%)	Pericarditis, cardiac tamponade, cardiomyopathy, arrhythmias, myocardial infarction, valvular disease (44%)
Renal involvement	Segmental necrotising glomerulonephritis with symptoms of nephritic syndrome, rare granulomatous features, abnormal urine tests (75%)	Segmental necrotising glomerulonephritis, reduced urine output, swelling in the lower extremities and face, elevated blood pressure, proteinuria (80–90%)	Segmental necrotising glomerulonephritis (40%)
Skin involvement	Palpable purpura (40%), cutaneous ulceration	Purpuric rash, nailed infarcts, splinter haemorrhages, livedo, ulceration (60%)	Palpable purpura (40–70%), cutaneous or subcutaneous nodules (30%)
Gastrointestinal involvement	Abdominal pain, diarrhoea, nausea, vomiting, bleeding (10%)	Abdominal pain, diarrhoea, gastrointestinal bleeding (30%)	Abdominal pain, diarrhoea, nausea, vomiting, bleeding (30%)
Nervous system involvement	Vasculitic neuropathy (10%)	Vasculitic neuropathy (30%)	Vasculitic neuropathy (80%)
Eye involvement	Orbital pseudotumour, scleritis (risk of scleromalacia perforans), episcleritis, uveitis (50%)	Scleritis, episcleritis, uveitis, optic neuropathy, orbital granulomata (20%)	Occasional eye disease: scleritis, episcleritis, uveitis, retinal vasculitis, exophthalmos
Diagnosis criteria	Typical histopathological pattern: characteristic pulmonary or urinary sediment changes, presence of PR3-ANCA	Typical clinical manifestations: typical histopathological pattern, presence of MPO-ANCA	Typical clinical manifestations: typical histopathological pattern, Lanham diagnostic criteria

**Table 3:** The association between ANCA staining patterns, targets, and particular diseases [78,81,82].

ANCA Staining Pattern	Target Antigens	Associated Diseases
C-ANCA	PR3	GPA > MPA Pauci-immune crescentic glomerulonephritis EGPA Infective endocarditis Amoeba infection Normal individual
P-ANCA	MPO > PR3 Other	MPA > EGPA > GPA Inflammatory bowel disease Cystic fibrosis Pauci-immune crescentic glomerulonephritis Primary sclerosing cholangitis Normal individual
A-ANCA	Multiple antigens	Inflammatory bowel disease Arthritis Drug-induced vasculitis Primary sclerosing cholangitis Autoimmune hepatitis Cocaine Hydralazine

## References

[1] Tsukui D., Kimura Y., Kono H. (2021). Pathogenesis and pathology of anti-neutrophil cytoplasmic antibody (ANCA)-associated vasculitis. J. Transl. Autoimmun..

[2] Jennette J., Falk R., Bacon P., Basu N., Cid M., Ferrario F., Flores-Suarez L., Gross W., Guillevin L., Hagen E. (2013). 2012 Revised International Chapel Hill Consensus Conference Nomenclature of Vasculitides. Arthritis Rheum..

[3] Segelmark M., Baslund B., Wieslander J. (1994). Some patients with anti-myeloperoxidase autoantibodies have a C-ANCA pattern. Clin. Exp. Immunol..

[4] Radice A., Bianchi L., Sinico R.A. (2013). Anti-neutrophil cytoplasmic autoantibodies: Methodological aspects and clinical significance in systemic vasculitis. Autoimmun. Rev..

[5] Cohen Tervaert J.W. (2019). Should proteinase-3 and myeloperoxidase anti-neutrophil cytoplasmic antibody vasculitis be treated differently: Part 2. Nephrol. Dial. Transpl..

[6] Watts R.A., Mahr A., Mohammad A.J., Gatenby P., Basu N., Flores-Suárez L.F. (2015). Classification, epidemiology and clinical subgrouping of antineutrophil cytoplasmic antibody (ANCA)-associated vasculitis. Nephrol. Dial. Transplant..

[7] Pearce F.A., Lanyon P.C., Grainge M.J., Shaunak R., Mahr A., Hubbard R.B., Watts R.A. (2016). Incidence of ANCA-associated vasculitis in a UK mixed ethnicity population. Rheumatology.

[8] Iudici M., Quartier P., Terrier B., Mouthon L., Guillevin L., Puéchal X. (2016). Childhood-onset granulomatosis with polyangiitis and microscopic polyangiitis: Systematic review and meta-analysis. Orphanet J. Rare Dis..

[9] Bataille P.M., Durel C.A., Chauveau D., Panes A., Thervet É.S., Terrier B. (2022). Epidemiology of granulomatosis with polyangiitis and microscopic polyangiitis in adults in France. J. Autoimmun..

[10] Redondo-Rodriguez R., Mena-Vázquez N., Cabezas-Lucena A.M., Manrique-Arija S., Mucientes A., Fernández-Nebro A. (2022). Systematic Review and Metaanalysis of Worldwide Incidence and Prevalence of Antineutrophil Cytoplasmic Antibody (ANCA) Associated Vasculitis. J. Clin. Med..

[11] Paroli M., Gioia C., Accapezzato D. (2023). New Insights into Pathogenesis and Treatment of ANCA-Associated Vasculitis: Autoantibodies and Beyond. Antibodies.

[12] Watts R.A., Hatemi G., Burns J.C., Mohammad A.J. (2021). Global epidemiology of vasculitis. Nat. Rev. Rheumatol..

[13] Runowska M.D., Majewski D. (2019). Ziarniniakowatość z zapaleniem naczyń-rys historyczny. Forum Reumatol..

[14] Langford C.A. (2001). Wegener Granulomatosis. Am. J. Med. Sci..

[15] Lamprecht P., Gross W.L. (2004). A brief history of Wegener’s granulomatosis: On limited, localized, and generalized forms of the disease: Comment on the article by the Wegener’s granulomatosis Etanercept Trial Research Group. Arthritis Rheum..

[16] Fahey J.L., Leonard E., Churg J., Godman G. (1954). Wegener’s Granulomatosis. Am. J. Med..

[17] McDonald J.B. (1960). “Wegener’s Granulomatosis”—A Triad. JAMA.

[18] Jennette J.C., Falk R.J. (2014). Pathogenesis of antineutrophil cytoplasmic autoantibody-mediated disease. Nat. Rev. Rheumatol..

[19] Jennette J.C., Falk R.J., Andrassy K., Bacon P.A., Churg J., Gross W.L., Hagen E.C., Hoffman G.S., Hunder G.G., Kallenberg C.G.M. (1994). Nomenclature of Systemic Vasculitides. Arthritis Rheum..

[20] Robson J.C., Grayson P.C., Ponte C., Suppiah R., Craven A., Judge A., Khalid S., Hutchings A., Watts R.A., Merkel P.A. (2022). 2022 American College of Rheumatology/European Alliance of Associations for Rheumatology classification criteria for granulomatosis with polyangiitis. Ann. Rheum. Dis..

[21] Luqmani R.A., Pathare S., Kwok-Fai T.L. (2005). How to diagnose and treat secondary forms of vasculitis. Best Pract. Res. Clin. Rheumatol..

[22] Peleg H., Ben-Chetrit E. (2017). Vasculitis in the autoinflammatory diseases. Curr. Opin. Rheumatol..

[23] Hewins P., Al-Abadi E. (2022). Medium vessel vasculitis. Medicine.

[24] Jennette J.C., Falk R.J. (1997). Small-Vessel Vasculitis. N. Engl. J. Med..

[25] Mahr A., Specks U., Jayne D. (2019). Subclassifying ANCA-associated vasculitis: A unifying view of disease spectrum. Rheumatology.

[26] Sunderkötter C., Golle L., Pillebout E., Michl C. (2023). Pathophysiology and clinical manifestations of immune complex vasculitides. Front. Med..

[27] Hendaoui L., Stanson A.W., Bouhaouala M.H., Joffre F. (2012). Systemic Vasculitis.

[28] Almaani S., Fussner L.A., Brodsky S., Meara A.S., Jayne D. (2021). ANCA-Associated Vasculitis: An Update. J. Clin. Med..

[29] Liew P.X., Kubes P. (2019). The Neutrophil’s Role During Health and Disease. Physiol. Rev..

[30] Coffelt S.B., Wellenstein M.D., de Visser K.E. (2016). Neutrophils in cancer: Neutral no more. Nat. Rev. Cancer.

[31] Summers C., Rankin S.M., Condliffe A.M., Singh N., Peters A.M., Chilvers E.R. (2010). Neutrophil kinetics in health and disease. Trends Immunol..

[32] Borges L., Pithon-Curi T.C., Curi R., Hatanaka E. (2020). COVID-19 and Neutrophils: The Relationship between Hyperinflammation and Neutrophil Extracellular Traps. Mediat. Inflamm..

[33] Silvestre-Roig C., Fridlender Z.G., Glogauer M., Scapini P. (2019). Neutrophil Diversity in Health and Disease. Trends Immunol..

[34] Evrard M., Kwok I.W., Chong S.Z., Teng K.W., Becht E., Chen J., Sieow J.L., Penny H.L., Ching G.C., Devi S. (2018). Developmental Analysis of Bone Marrow Neutrophils Reveals Populations Specialized in Expansion, Trafficking, and Effector Functions. Immunity.

[35] Hidalgo A., Chilvers E.R., Summers C., Koenderman L. (2019). The Neutrophil Life Cycle. Trends Immunol..

[36] Nandakumar S.K., Ulirsch J.C., Sankaran V.G. (2016). Advances in understanding erythropoiesis: Evolving perspectives. Br. J. Haematol..

[37] Tang X., Xu Q., Yang S., Huang X., Wang L., Huang F., Luo J., Zhou X., Wu A., Mei Q. (2023). Toll-like Receptors and Thrombopoiesis. Int. J. Mol. Sci..

[38] Hong C.W. (2017). Current Understanding in Neutrophil Differentiation and Heterogeneity. Immune Netw..

[39] Cowland J.B., Borregaard N. (2016). Granulopoiesis and granules of human neutrophils. Immunol. Rev..

[40] Özcan A., Boyman O. (2022). Mechanisms regulating neutrophil responses in immunity, allergy, and autoimmunity. Allergy.

[41] Aratani Y. (2018). Myeloperoxidase: Its role for host defense, inflammation, and neutrophil function. Arch. Biochem. Biophys..

[42] Kallenberg C.G. (2008). Pathogenesis of PR3-ANCA Associated Vasculitis. J. Autoimmun..

[43] Néel A., Degauque N., Bruneau S., Braudeau C., Bucchia M., Caristan A., Mornac D.D., Genin V., Glemain A., Oriot C. (2022). Pathogénie des vascularites associées aux ANCA en 2021: Mise au point. Rev. Méd. Interne.

[44] Nakazawa D., Masuda S., Tomaru U., Ishizu A. (2018). Pathogenesis and therapeutic interventions for ANCA-associated vasculitis. Nat. Rev. Rheumatol..

[45] Sun X.J., Li Z.Y., Chen M. (2023). Pathogenesis of anti-neutrophil cytoplasmic antibody-associated vasculitis. Rheumatol. Immunol. Res..

[46] Merino-Vico A., van Hamburg J.P., Tas S.W. (2021). B Lineage Cells in ANCA-Associated Vasculitis. Int. J. Mol. Sci..

[47] d’Alessandro M., Conticini E., Bergantini L., Cameli P., Cantarini L., Frediani B., Bargagli E. (2022). Neutrophil Extracellular Traps in ANCA-Associated Vasculitis and Interstitial Lung Disease: A Scoping Review. Life.

[48] Schreiber A., Rousselle A., Becker J.U., von Mässenhausen A., Linkermann A., Kettritz R. (2017). Necroptosis Controls NET Generation and Mediates Complement Activation, Endothelial Damage, and Autoimmune Vasculitis. Proc. Natl. Acad. Sci. USA.

[49] Kimura H., Matsuyama Y., Araki S., Koizumi A., Kariya Y., Takasuga S., Eguchi S., Nakanishi H., Sasaki J., Sasaki T. (2018). The effect and possible clinical efficacy of in vivo inhibition of neutrophil extracellular traps by blockade of PI3K-gamma on the pathogenesis of microscopic polyangiitis. Mod. Rheumatol..

[50] Suire S., Condliffe A.M., Ferguson G.J., Ellson C.D., Guillou H., Davidson K., Welch H., Coadwell J., Turner M., Chilvers E.R. (2006). Gbetagammas and the Ras binding domain of p110gamma are both important regulators of PI(3)Kgamma signalling in neutrophils. Nat. Cell Biol..

[51] Schreiber A., Xiao H., Jennette J.C., Schneider W., Luft F.C., Kettritz R. (2009). C5a receptor mediates neutrophil activation and ANCA-induced glomerulonephritis. J. Am. Soc. Nephrol..

[52] Uozumi R., Iguchi R., Masuda S., Nishibata Y., Nakazawa D., Tomaru U., Ishizu A. (2019). Pharmaceutical immunoglobulins reduce neutrophil extracellular trap formation and ameliorate the development of MPO-ANCA-associated vasculitis. Mod. Rheumatol..

[53] Kühnle A., Veelken R., Galuska C.E., Saftenberger M., Verleih M., Schuppe H.C., Rudloff S., Kunz C., Galuska S.P. (2019). Polysialic acid interacts with lactoferrin and supports its activity to inhibit the release of neutrophil extracellular traps. Carbohydr. Polym..

[54] Okubo K., Kamiya M., Urano Y., Nishi H., Herter J.M., Mayadas T., Hirohama D., Suzuki K., Kawakami H., Tanaka M. (2016). Lactoferrin Suppresses Neutrophil Extracellular Traps Release in Inflammation. eBioMedicine.

[55] Hidalgo A., Libby P., Soehnlein O., Aramburu I.V., Papayannopoulos V., Silvestre-Roig C. (2021). Neutrophil extracellular traps: From physiology to pathology. Cardiovasc. Res..

[56] Yoshida M., Yamada M., Sudo Y., Kojima T., Tomiyasu T., Yoshikawa N., Oda T., Yamada M. (2016). Myeloperoxidase anti-neutrophil cytoplasmic antibody affinity is associated with the formation of neutrophil extracellular traps in the kidney and vasculitis activity in myeloperoxidase anti-neutrophil cytoplasmic antibody-associated microscopic polyangiitis. Nephrology.

[57] Sangaletti S., Tripodo C., Chiodoni C., Guarnotta C., Cappetti B., Casalini P., Piconese S., Parenza M., Guiducci C., Vitali C. (2012). Neutrophil Extracellular Traps Mediate Transfer of Cytoplasmic Neutrophil Antigens to Myeloid Dendritic Cells Toward ANCA Induction and Associated Autoimmunity. Blood.

[58] Grayson P.C., Kaplan M.J. (2016). At the Bench: Neutrophil Extracellular Traps (NETs) Highlight Novel Aspects of Innate Immune System Involvement in Autoimmune Diseases. J. Leukoc. Biol..

[59] Ma Y., Yang X., Chatterjee V., Meegan J.E., Beard R.S., Yuan S.Y. (2019). Role of Neutrophil Extracellular Traps and Vesicles in Regulating Vascular Endothelial Permeability. Front. Immunol..

[60] Wilde B., van Paassen P., Witzke O., Tervaert J.W.C. (2011). New pathophysiological insights and treatment of ANCA-associated vasculitis. Kidney Int..

[61] Shi L. (2017). Anti-neutrophil cytoplasmic antibody-associated vasculitis: Prevalence, treatment, and outcomes. Rheumatol. Int..

[62] Wallace Z.S., Miloslavsky E.M. (2020). Management of ANCA associated vasculitis. BMJ.

[63] Quintana L.F., Kronbichler A., Blasco M., Zhao M.H., Jayne D. (2019). ANCA associated vasculitis: The journey to complement-targeted therapies. Mol. Immunol..

[64] Sakai R., Kondo T., Kurasawa T., Nishi E., Okuyama A., Chino K., Shibata A., Okada Y., Takei H., Nagasawa H. (2017). Current clinical evidence of tocilizumab for the treatment of ANCA-associated vasculitis: A prospective case series for microscopic polyangiitis in a combination with corticosteroids and literature review. Clin. Rheumatol..

[65] Monogioudi E., Sheldon J., Meroni P.L., Hutu D.P., Schimmel H., Zegers I. (2019). Certified reference material against PR3 ANCA IgG autoantibodies. From development to certification. Clin. Chem. Lab. Med..

[66] Gapud E., Seo P., Antiochos B. (2017). ANCA-Associated Vasculitis Pathogenesis: A Commentary. Curr. Rheumatol. Rep..

[67] van der Geest K.S.M., Brouwer E., Sanders J.S., Sandovici M., Bos N.A., Boots A.M.H., Abdulahad W.H., Stegeman C.A., Kallenberg C.G.M., Heeringa P. (2017). Towards precision medicine in ANCA-associated vasculitis. Rheumatology.

[68] Cornec D., Gall E.C.L., Fervenza F.C., Specks U. (2016). ANCA-associated vasculitis—Clinical utility of using ANCA specificity to classify patients. Nat. Rev. Rheumatol..

[69] Franssen C.F.M., Huitema M.G., Kobold A.C.M., Oost-Kort W.W., Limburg P.C., Tiebosch A., Stegeman C.A., Kallenberg C.G.M., Tervaert J.W.C. (1999). In Vitro Neutrophil Activation by Antibodies to Proteinase 3 and Myeloperoxidase from Patients with Crescentic Glomerulonephritis. J. Am. Soc. Nephrol..

[70] Moodie F.D., Leaker B., Cambridge G., Totty N.F., Segal A.W. (1993). Alpha-enolase: A Novel Cytosolic Autoantigen in ANCA Positive Vasculitis. Kidney Int..

[71] Yu F., Chen M., Gao Y., Wang S.X., Zou W.Z., Zhao M.H., Wang H.Y. (2007). Clinical and Pathological Features of Renal Involvement in Propylthiouracil-Associated ANCA-Positive Vasculitis. Am. J. Kidney Dis..

[72] Suzuki K., Nagao T., Itabashi M., Hamano Y., Sugamata R., Yamazaki Y., Yumura W., Tsukita S., Wang P.C., Nakayama T. (2014). A Novel Autoantibody Against Moesin in the Serum of Patients with MPO-ANCA-Associated Vasculitis. Nephrol. Dial. Transplant..

[73] Nagao T., Suzuki K., Utsunomiya K., Matsumura M., Saiga K., Wang P.C., Minamitani H., Aratani Y., Nakayama T., Suzuki K. (2011). Direct Activation of Glomerular Endothelial Cells by Anti-Moesin Activity of Anti-Myeloperoxidase Antibody. Nephrol. Dial. Transplant..

[74] Fukuhara A., Tanino Y., Sato S., Ishii T., Nikaido T., Kanazawa K., Saito J.I., Ishida T., Kanno M., Watanabe T. (2013). Systemic Vasculitis Associated with Anti-Neutrophil Cytoplasmic Antibodies Against Bactericidal/Permeability Increasing Protein. Intern. Med..

[75] Guchelaar N.A., Waling M.M., Adhin A.A., van Daele P.L., Schreurs M.W., Rombach S.M. (2021). The value of anti-neutrophil cytoplasmic antibodies (ANCA) testing for the diagnosis of ANCA-associated vasculitis, a systematic review and meta-analysis. Autoimmun. Rev..

[76] Csernok E. (2018). New concepts in ANCA detection and disease classification in small vessel vasculitis: The role of ANCA antigen specificity. Mediterr. J. Rheumatol..

[77] Dalpé G., Fernandes F., Richard C., Boire G., Ménard H.A. (1993). Heterogeneity of ANCA Sera Showing Atypical, Peripheral and Classical Cytoplasmic Immunofluorescence Patterns. Advances in Experimental Medicine and Biology.

[78] van Beers J.J.B.C., Vanderlocht J., Roozendaal C., Damoiseaux J. (2018). Detection of Anti-neutrophil Cytoplasmic Antibodies (ANCA) by Indirect Immunofluorescence. Autoantibodies.

[79] Moiseev S., Tervaert J.W.C., Arimura Y., Bogdanos D.P., Csernok E., Damoiseaux J., Ferrante M., Flores-Suárez L.F., Fritzler M.J., Invernizzi P. (2020). 2020 international consensus on ANCA testing beyond systemic vasculitis. Autoimmun. Rev..

[80] Savige J., Gillis D., Benson E., Davies D., Esnault V., Falk R.J., Hagen E.C., Jayne D., Jennette J.C., Paspaliaris B. (1999). International Consensus Statement on Testing and Reporting of Antineutrophil Cytoplasmic Antibodies (ANCA). Am. J. Clin. Pathol..

[81] Suwanchote S., Rachayon M., Rodsaward P., Wongpiyabovorn J., Deekajorndech T., Wright H.L., Edwards S.W., Beresford M.W., Rerknimitr P., Chiewchengchol D. (2018). Anti-neutrophil cytoplasmic antibodies and their clinical significance. Clin. Rheumatol..

[82] Savige J., Trevisin M., Pollock W. (2018). Testing and reporting antineutrophil cytoplasmic antibodies (ANCA) in treated vasculitis and non-vasculitic disease. J. Immunol. Methods.

[83] Kempiners N., Mahrhold J., Hellmich B., Csernok E. (2020). Evaluation of PR3- and MPO-ANCA line and dot immunoassays in ANCA-associated vasculitis. Rheumatology.

[84] Csernok E., Damoiseaux J., Rasmussen N., Hellmich B., van Paassen P., Vermeersch P., Blockmans D., Tervaert J.W.C., Bossuyt X. (2016). Evaluation of automated multi-parametric indirect immunofluorescence assays to detect anti-neutrophil cytoplasmic antibodies (ANCA) in granulomatosis with polyangiitis (GPA) and microscopic polyangiitis (MPA). Autoimmun. Rev..

[85] Shovman O., Agmon-Levin N., Gilburd B., Martins T., Petzold A., Matthias T., Shoenfeld Y. (2014). A fully automated IIF system for the detection of antinuclear antibodies and antineutrophil cytoplasmic antibodies. Immunol. Res..

[86] Damoiseaux J., Csernok E., Rasmussen N., Moosig F., van Paassen P., Baslund B., Vermeersch P., Blockmans D., Tervaert J.W.C., Bossuyt X. (2016). Detection of antineutrophil cytoplasmic antibodies (ANCAs): A multicentre European Vasculitis Study Group (EUVAS) evaluation of the value of indirect immunofluorescence (IIF) versus antigen-specific immunoassays. Ann. Rheum. Dis..

[87] Renuncio-García M., Calvo-Río V., Benavides-Villanueva F., Fazazi S.A., Rodríguez-Vidriales M., Escagedo-Cagigas C., Martín-Penagos L., Irure-Ventura J., López-Hoyos M., Blanco R. (2023). ANCA detection with solid phase chemiluminescence assay: Diagnostic and severity association in vasculitis. Immunol. Res..

[88] Bossuyt X., Tervaert J.W.C., Arimura Y., Blockmans D., Flores-Suárez L.F., Guillevin L., Hellmich B., Jayne D., Jennette J.C., Kallenberg C.G.M. (2017). Revised 2017 international consensus on testing of ANCAs in granulomatosis with polyangiitis and microscopic polyangiitis. Nat. Rev. Rheumatol..

[89] Baggiolini M., Bretz U., Dewald B., Feigenson M.E. (1978). The Polymorphonuclear Leukocyte. Agents Actions.

[90] Sturrock A.B., Espinosa R.I., Hoidal J.R., Le Beau M.M. (1993). Localization of the Gene Encoding Proteinase-3 (the Wegener’s Granulomatosis Autoantigen) to Human Chromosome Band 19p13.3. Cytogenet. Cell Genet..

[91] Gencik M., Meller S., Borgmann S., Fricke H. (2000). Proteinase 3 Gene Polymorphisms and Wegener’s Granulomatosis. Kidney Int..

[92] Specks U. (2009). Epitope-specific anti-neutrophil cytoplasmic antibodies: Do they matter? Can they be detected?. APMIS.

[93] Hunter R.W., Welsh N., Farrah T.E., Gallacher P.J., Dhaun N. (2020). ANCA associated vasculitis. BMJ.

[94] Basu N., Watts R., Bajema I., Baslund B., Bley T., Boers M., Brogan P., Calabrese L., Cid M.C., Cohen-Tervaert J.W. (2010). EULAR points to consider in the development of classification and diagnostic criteria in systemic vasculitis. Ann. Rheum. Dis..

[95] Suresh E. (2006). Diagnostic approach to patients with suspected vasculitis. Postgrad. Med. J..

[96] Guggenberger K.V., Bley T.A. (2020). Imaging in Vasculitis. Curr. Rheumatol. Rep..

[97] Gaffo A.L. (2010). Diagnostic Approach to ANCA-associated Vasculitides. Rheum. Dis. Clin. N. Am..

[98] Damoiseaux J. (2021). ANCA testing in clinical practice: From implementation to Quality Control and Harmonization. Front. Immunol..

[99] Sinico R.A., Radice A. (2014). Antineutrophil cytoplasmic antibodies (ANCA) testing: Detection methods and clinical application. Clin. Exp. Rheumatol..

